# Watch out for the special location of intraventricular silicone oil following an intraocular tamponade - a 10-year follow-up case report based on CT/MRI

**DOI:** 10.1186/s12886-019-1286-8

**Published:** 2019-12-30

**Authors:** Juntao Cao, Lianlong Bian, Pengpeng Zhou, Jianchun Tu

**Affiliations:** 1 Department of Radiology, Kunshan Affiliated Hospital of Nanjing University of Chinese Medicine, 189 Chaoyangxi Road, Kunshan, 215300 Jiangsu China; 2 Department of Ophthalmology, Kunshan Affiliated Hospital of Nanjing University of Chinese Medicine, 189 Chaoyangxi Road,Kunshan,215300 Jiangsu, China

**Keywords:** Silicone oil, Intraocular, Intraventricular, CT, MRI

## Abstract

**Background:**

Intraventricular silicone oil is a relatively rare complication resulted from silicone oil tamponade to treat retinal detachment. It is occasionally reported in previous literature. To the best of our knowledge, the long-term longitudinal comparisons of silicone oil both in the brain and in the postoperative eyeball based on CT/MRI were lacking, and intraventricular silicone oil accumulation beside lesions has been reported rarely.

**Case presentation:**

A 63-year-old male patient underwent an intraocular tamponade with silicone oil in June 2009. Eight CT examinations and 2 MRI examinations were acquired between 2011 and 2018.The changes of silicone oil in the brain in CT/MRI as below: Silicone oil initially migration to bilateral lateral ventricular anterior horn was found in November 2011, it was aslo found at right side of suprasellar cisterna, and there was no change in location 6 h later; Silicone oil at the anterior horn of right lateral ventricle disappeared but remained at left lateral ventricle and right side of suprasellar cisterna in July 2014, and there was no change in location in a short-term reexamination. It was found at the middle of left lateral ventricle (adjacent to the real cause) in march 2018, but disappeared 3 months later, while remained at anterior horn of left lateral ventricular and right side of suprasellar cisterna all the time. There was no change in location in the next 2 follow-up (September and October in 2018). The CT values of silicone oil distributed throughout the brain were dynamically changed with time.

**Conclusion:**

It is important to recognize intraventricular silicone oil in a particular location.More important is to discover “the real murderer”, which is the main cause of symptoms in the vicinity of special location. Moreover, the migration of silicone oil between eyeball and brain may not be always in a single direction.

## Background

Silicone oil has been used as a vitreous filler to treat retinal detachment for over half a century with safety and stability [1, 2], and the main complications are including oil cysts in conjunctiva, banded keratopathy, and secondary glaucoma, and even vision loss caused by optic nerve and retinal damage [3–5]. Silicone oil leakage and migration to the brain is occasionally reported in existing literature and it is usually asymptomatic [6–8]. However, to the best of our knowledge, it is rare to report CT/MRI long-term longitudinal comparisons of silicone oil both in the brain and in the postoperative eyeball.

## Case presentation

In this study, a 63-year-old male patient was presented, who had undergone silicone oil tamponade in the right eyeball in June 2009 as a result of diabetic complications. No significant discomfort was observed at the initial stage postoperatively, but unfortunately, the right eye vision decreased gradually on the 2nd month, and completely blind on the 3rd month after operation. The silicone oil in the right eye was not removed for maintaining the normal eyeball in morphology.

**Fig. 1:**
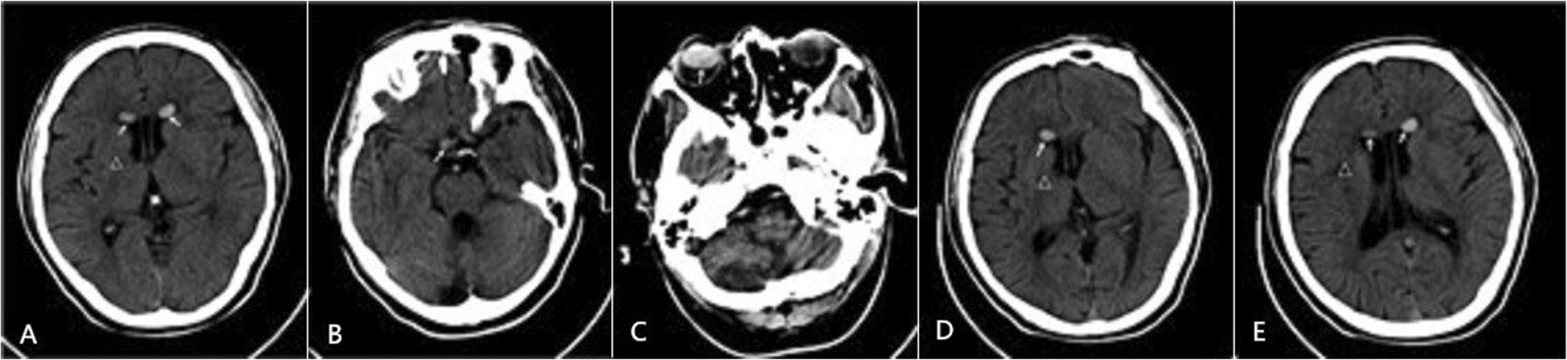
A 63-year-old male undergoes CT examination in December 2011 (ww 60, wl 100). **a** ~ **c** show the high-density shadow at bilateral ventricular anterior horns, right of suprasellar cistern and right eyeball, respectively (arrow). **d** and **e** display that the location of the high-density shadow in bilateral lateral ventricular anterior horns remains unchanged after 6 h (arrow).The old infarction at right basal ganglia is shown in **a**, **d** and **e** (△)

In November 2011, the patient came to see a doctor because of dizziness and headache.an emergency CT examination had occasionally shown the quasi-circular high-density shadows at bilateral lateral ventricular anterior horns, and a small high-density shadow at the right side of suprasellar cistern; besides, similar findings were also discovered in the right eyeball in addition to the low-density regions at the right side of basal ganglia and corona radiata. Therefore, the patient was suspected with old cerebral infarction at right side of basal ganglia/corona radiata, and cerebral hemorrhage was not excluded. Moreover, the high-density lesions were still found at bilateral lateral ventricular anterior horns and at the right side of suprasellar cistern on the re-examined CT 6 h later. A similar finding was also found in the right optic nerve, which is thicker and denser than the left. Then the diagnosis of intraventricular migration of silicone oil after an intraocular tamponade was made based on the patient’s medical history (Fig. 1).The cause of the patient’s symptoms is considered to be hypertension, sequelae of cerebral infarction, and the patient is also accompanied by diabetes. The patient’s symptoms improved markedly through a series of symptomatic treatments, including nutritional support, adjust blood pressure, and control blood sugar.

**Fig. 2:**
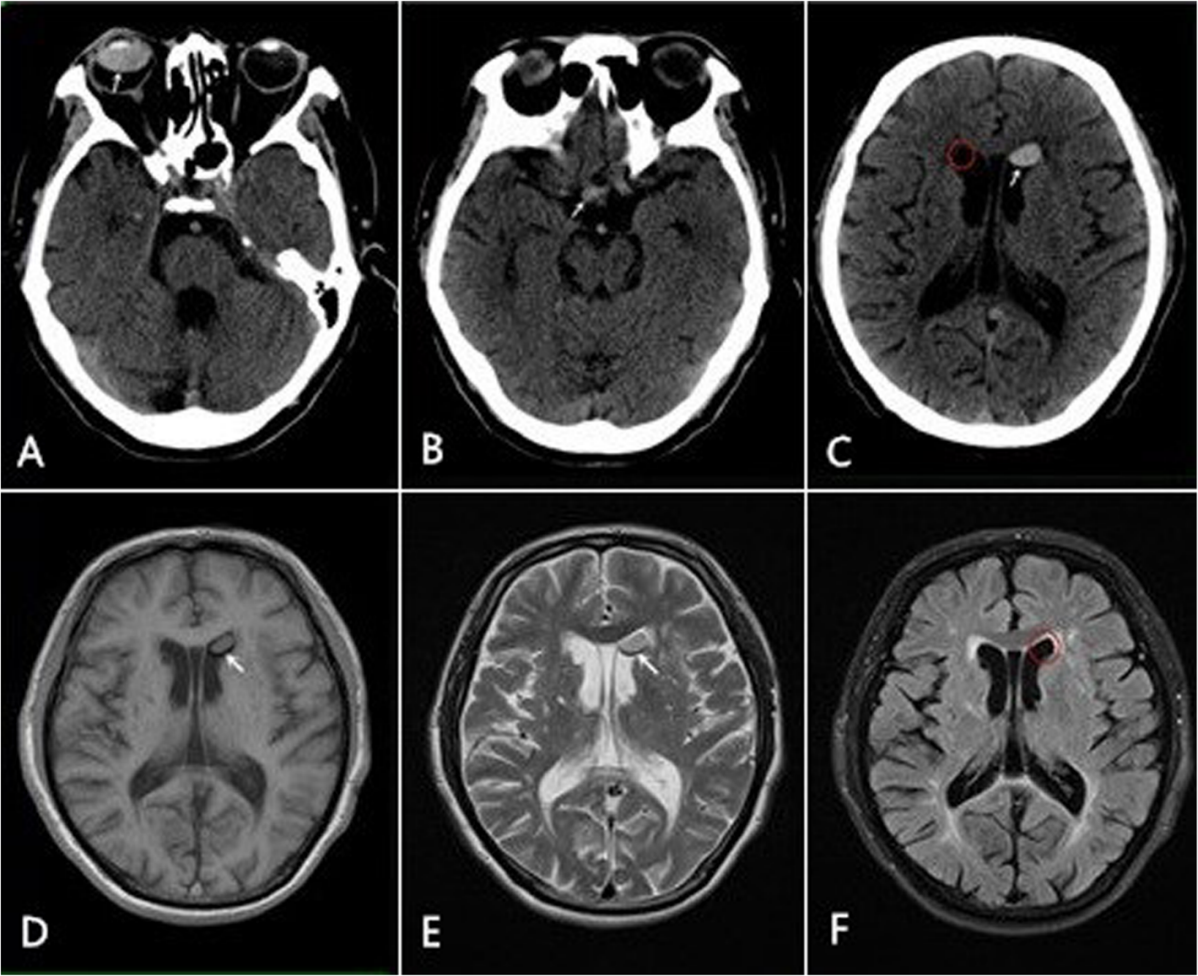
The patient receives CT and MRI examinations in July 2014. **a**~**b**, the high or slightly high density shadow on the right side of the right eyeball and the right suprasellar cistern (ww 60, wl 100)(arrow); **c**, the high-density shadow on the left ventricular anterior horn (arrow), disappearance of the high-density shadow on the right ventricular anterior horn (red circle) (ww/wl same as A); **d**~**e**, lesions with slightly high signal intensities on T1WI and T2WI, and chemical shift artifact on the edge of lesion (arrow); **f**, disappearance of the slightly high signal intensity on T2WI_tirm_dark-fluid (red circle)

In July 2014, the patient was hospitalized due to dizziness and walking instability.A CT examination revealed the greatly decreased density of bilateral cerebella and brainstems, suggesting cerebral infarction (as subsequently confirmed by MRI). At the same time, the high-density lesion at the anterior horn of right lateral ventricle had disappeared. However, the quasi-circular high-density shadow could still be observed at the anterior horn of the left lateral ventricle, along with the small high-density shadow in the right side of suprasellar cistern. No significant change was detected at the anterior lesion of the left lateral ventricle 3 days later, while the density of lesion at the right side of suprasellar cistern was slightly increased. Afterwards, the patient had received craniocerebral MRI examination, which revealed that the lesion at left lateral ventricle anterior horn had displayed the consistent signal characteristics as those in right eyeball, including slightly higher signal on T1WI (on which water appears as low signal and lipid components as slightly higher signal), slightly higher signal on T2WI(on which water appears as high signal and lipid components as slightly higher signal), chemical shift artifacts (a special artifact that is easy to appear on the surface of fat in MRI) on both T1WI and T2WI, low signal on T2WI lipid-pressing image (Fig. 2).The main cause of symptoms in this patient is subacute cerebral infarction in the brain.The patient’s symptoms improved significantly with a standard procedure for stroke therapy in neurology, including thrombolysis, improvement of brain metabolism, nutritional support, while controlling blood pressure and blood sugar.

**Fig. 3:**
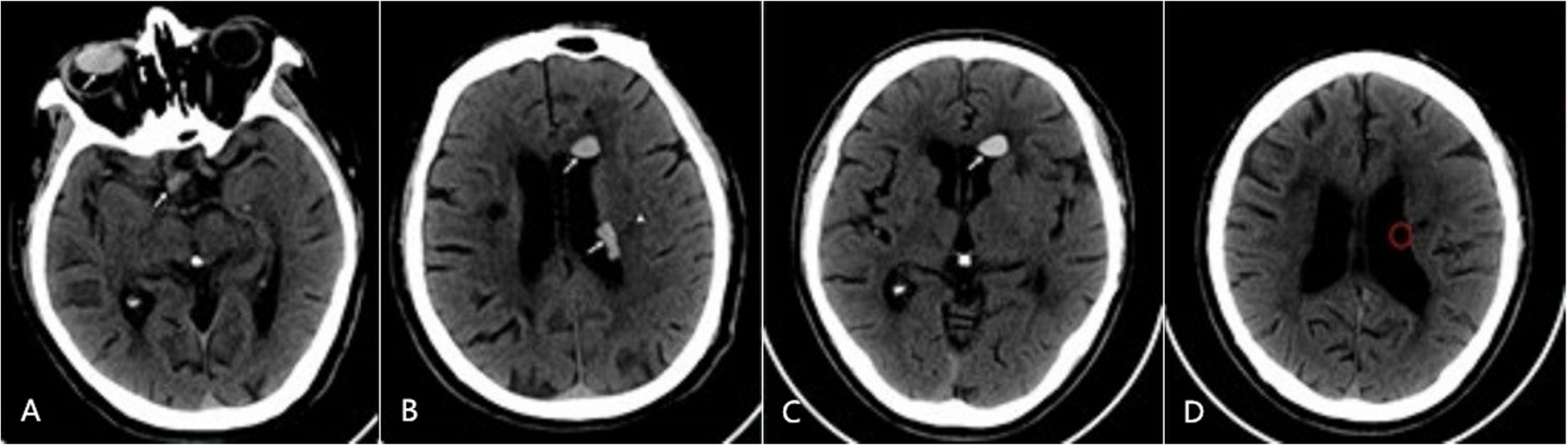
The patient undergoes CT examinations in March and June 2018. **a**, the high or slightly high density shadows in the right eyeball and the right suprasellar cistern (ww 60, wl 100)(arrow); **b**, the high density shadow at anterior horn and middle of the left lateral ventricle (ww/wl same as A)(arrow), the slightly low density shadow at the left corona radiata (arrowed), prompting infarction (subsequently confirmed by MRI); **c**~**d**, CT reexamination after 3 months shows that the lesion at the left lateral ventricular anterior horn has a higher density and a larger volume (ww/wl same as A) (arrow), but it disappears in the middle of left lateral ventricle (ww/wl same as A)(red circle)

In March 2018, the patient was re-admitted as a result of cerebral infarction symptoms with unknown cause. A CT examination showed that the quasi-circular high-density lesions in the right eyeball and the anterior horn of the left lateral ventricle still existed, and the high-density shadow at the right side of suprasellar cistern remained almost unchanged. However, a new strip-shaped high-density lesion was found at the middle of left lateral ventricle, which was well-defined on the ventricular side, but ill-defined on another side adjacent to the left corona radiata, where a slightly low-density lesion was also discovered (subsequently confirmed by MRI as a new cerebral infarction). MRI examination revealed that the lesions both at middle and anterior of left lateral ventricle had exhibited the same signal characteristics as most of those obtained previously (2014.07). Additionally, There were some new change observed. The ADC maps (on which water molecule diffusion movement visual display; the more restricted the motion, the lower the signal) showed that the signal of silicone oil at left lateral ventricle (anterior horn and middle) were more complicated than those obtained in 2014, and it had more dotted iso-hyper signals compared to the former. Three months later, the re-examined CT suggested that the high-density lesion in the middle of the left lateral ventricle had disappeared, but the quasi-circular high-density lesion could still be detected in the anterior horn of left lateral ventricle; meanwhile, the density of silicone oil elsewhere increased. We found that the high-density lesion had persisted in the anterior horn of left lateral ventricle in the next two CT reexaminations (in 2018.09 and 2018.10), while the high-density shadow in the right suprasellar cistern had displayed a slight sustained increase in density (Fig. 3). The patient underwent an active thrombolytic therapy, but the effect of intervention on imaging founding was not obvious compared to the improvement of his symptoms.

**Fig. 4:**
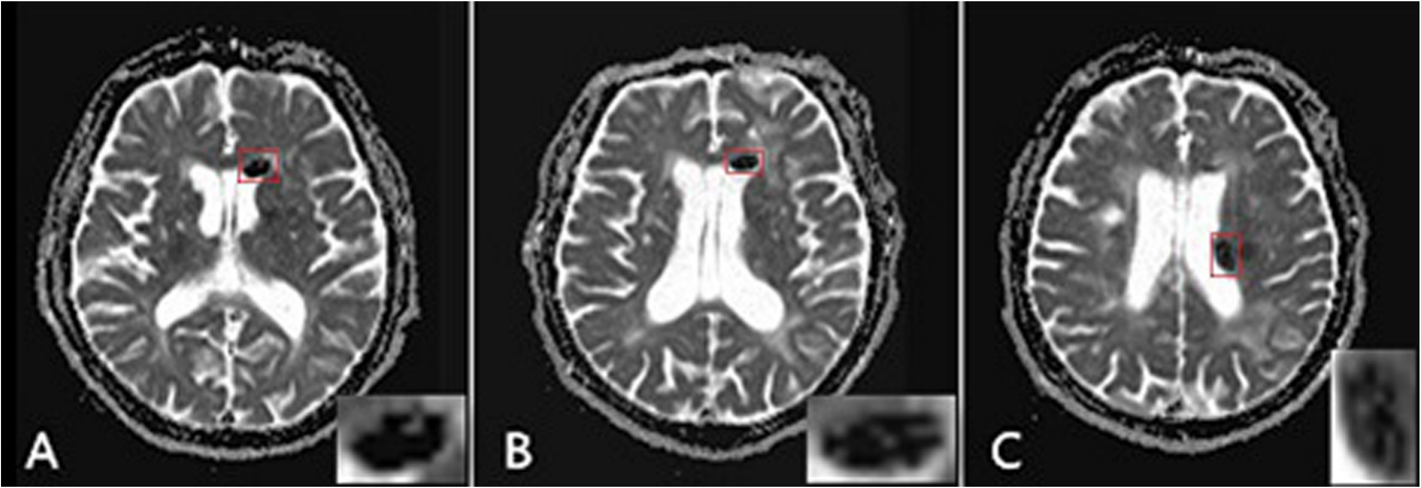
The ADC maps of ventricular silicone oils acquired in 2014 (**a**) and 2018 (**b**, **c**).The internal signals of these lesions are not uniform (red boxes, enlarged in the lower right corner), but the signals (**b**, **c**) are more mixed compared with the former (**a**)

**Fig. 5:**
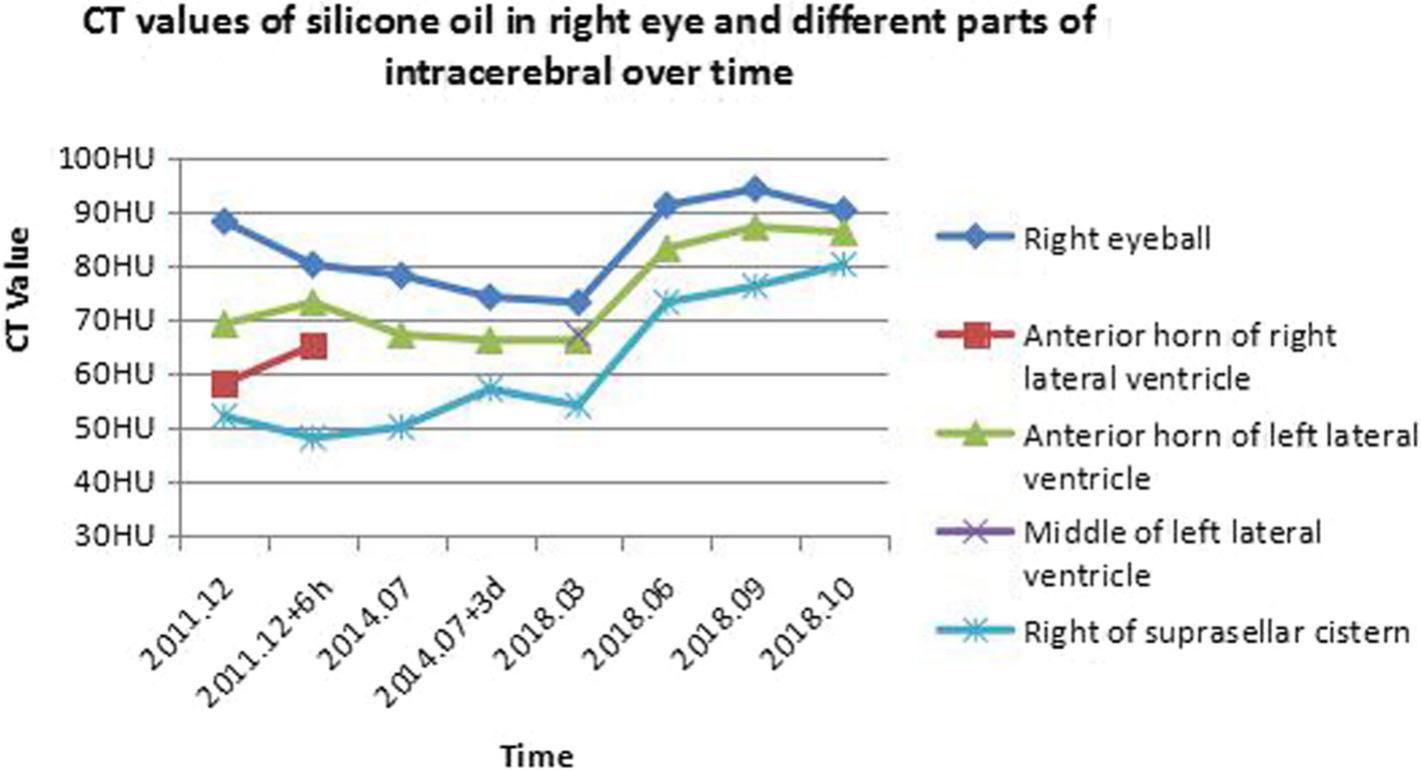
CT values of silicone oil in right eye and brain are constantly changing with time. The increase in density of silicone oil in one place is always accompanied by the decrease in density of silicone oil or disappearance elsewhere

In addition, the ADC maps of lateral ventricular silicone oil acquired in 2014 and 2018 had revealed some subtle changes (Fig. 4).The CT values (reflecting material density, HU as its unit) of silicone oil in the right eyeball and in different intracerebral parts over time were shown in Fig. 5.

## Discussion and conclusion

According to literature report, intracerebral migration of silicone oil may take place from 2 months to 12 years after the initial intraocular endotamponade with silicone oil [6, 9]. Extraocular silicone oil is mainly distributed in subconjunctival space, optic nerve, optic chiasm, lateral ventricle, as well as the third and the fourth ventricles [3, 7, 10, 11]. Specifically, most of the silicone oils are found accidentally, and they are relatively fixed in unrestricted locations; however, long-term tracking records of intraventricular silicone oil are lacking. It is clinically unclear about whether silicone oil that migrates to extraocular region should be treated surgically or not, and the patients are usually asymptomatic. In our case, ventricular migration of silicone oil was found at 30th month postoperatively, but the initial migration might be earlier. The patient was followed up at different intervals, and the imaging characteristics of intraventricular silicone oil were recorded at various time periods. Our research suggested that the morphology or density/signal intensity of intraventricular silicone oil was changing inconstantly, which had displayed a process of migration, aggregation, separation and re-aggregation. In addition, the density of intraocular silicone oil had undergone impressive changes after surgery, which had a CT value range from 73HU to 94HU (one thousandth of the difference between water and air in density as one HU).

The mechanism of silicone oil migration to the subarachnoid space or ventricle remains unclear so far, although it has been discussed in many studies from the point of pathophysiology or anatomy [11, 12].Here are some local risk factors that are likely to cause leakage of intraocular silicone oil:1 silicone oil emulsification, the emulsified silicone oil can be easily separated into small droplets and migrate out of the eyeball, which is related to the silicone oil filling time, silicone oil molecular weight and application of surfactants during perioperative period [4]; 2 increased intraocular pressure, which is considered to be the main factor leading to migration, but not a separate one [13];3 pre-existing glaucoma or abnormalities of the optic nerve and intraocular structure, such as optic pits, internal limiting membrane incomplete, and etc. [6, 14];4 changes in local inner environment and macrophage phagocytosis of emulsified oil bubbles, which may be the potential migration factors [9]. As discovered from this case report and other literature reviews, the migration pathway of intraocular silicone oil might be shown below: silicone oil droplets in vitreous cavity could migrate to the retina and/or the optic nerve sheath through the fissures, which could then enter the intracranial regions along the optic nerve sheath and the optic chiasm, including lateral ventricle, the third and the fourth ventricles [6, 7, 10, 14–16]. However, It is not clear where the silicone oil will eventually migrate to.

The imaging findings of intraventricular silicone oil were basically consistent with those reported previously, which included high density on CT, slightly high signal on T1WI, and slightly high signal on T2WI, accompanying with chemical shift artifacts, as well as markedly low signal on T1WI or T2WI lipid suppression images (on which the lipid components could be easily identified) [11, 16, 17]. We noticed that the silicone oil both in intraocular and brain were changing in terms of CT density and MRI signal intensity (as shown in Fig. 5). The ADC maps suggested that, the above changes were partially determined by accumulating degree of silicone oil droplets in cerebrospinal fluid (CSF), which could also confirm the separation and reunification process of silicone oil in the ventricle. It is temporarily called “small steps”, which can also explain its difficulty to detect a small amount of silicone oil migration on CT/MRI image. Typically, the body position and specific gravity of silicone oil are the key factors determining this process. Silicone oil gengrally used has a slightly lower specific gravity than that of CSF [18]. Normal ventricular walls are smooth, and silicone oil can accumulate in the non-dependent part of the ventricle, so it is always located at the apical ventricle.

However, why did the silicone oil stay in the middle of left lateral ventricle for a period of time in this case? The key factor was the new cerebral infarction in left radiation corona, which made local ependyma non-smooth and restricted the movement of silicone oil.The silicone oil-water clusters were more likely to stop at the unsmooth area of ependyma. In theory, the migration location of intraventricular silicone oil depends on patient position and CSF circulation, which may be the cistern magnum, spinal subdural space, and extraspinal canal through the intervertebral foramen, or even the surgical eyeball. Typically, intraventricular silicone oil is not invariable in the brain, instead, it will change with time, position and other factors affecting its movement. It could be seen from Fig. 5 that the increased density of silicone oil in brain always be accompanied by the decreased density or disappeared of another silicone oil. Finally, due to the lacking of sufficient volume data, we were unable to accurately assess the volume of silicone oil in the brain. Further study will benefit from MRI 3D sequences (e.g. 3D-T1WI, from which the precise volume of silicone oil in the brain can be obtained).

It is important to recognize the extraocular silicone oil, especially in brain in emergency circumstance; more importantly, it is also of vital significance to recognize whether the silicone oil stays in the unrestricted areas of the brain or not, and the occurrence of other lesions may be alerted. This requires to understand the detailed medical history, recognize its imaging characteristics, prevent the covering of real lesions and identify the” real murderers”, including intracerebral hemorrhage, metastasis, lymphoma, choroid plexus papilloma/carcinoma, meningioma, subependymoma, ependymoma and etc. Meanwhile, the migration of silicone oil between eyeball and brain may be bidirectional, which may provide an alternative to the removal of silicone oil in the brain compared to surgery(if necessary).

## Data Availability

The datasets used and analysed during the current study are available from the corresponding author on reasonable request.

## Abbreviations

ADC: Apparent diffusion coefficient
CT: Computed tomography
HU: Hounsfield Unit
MRI: Magnetic resonance imaging
T1WI: T1 weighted image
T2WI: T2 weighted image
wl: window level
ww: window width
